# Acute monolateral proptosis and orbital myositis in a patient with discoid lupus erythematosus: a case report

**DOI:** 10.1186/1752-1947-8-375

**Published:** 2014-11-20

**Authors:** Loredana Arrico, Alessandro Abbouda, Simona Bianchi, Romualdo Malagola

**Affiliations:** 1 Department of Ophthalmology, University of Rome, Sapienza Viale del Policlinico 155, 00186 Roma, Italy

**Keywords:** Computed tomography, Discoid lupus erythematosus, Orbital myositis, Ultrasound echography

## Abstract

**Introduction:**

Discoid lupus erythematosus may lead to significant orbital inflammation syndrome. Ocular manifestations related to discoid lupus erythematosus are uncommon and few cases of eye inflammation are reported.

**Case presentation:**

A 37-year-old Caucasian woman with 5-year history of discoid lupus erythematosus presented with exophthalmos, periorbital pain and blurred vision in her right eye. Orbital computed tomography and laboratory tests were performed. Computed tomography imaging revealed an enlargement of the right medial rectus muscle. Thyroid eye disease and orbital cellulites were excluded. Corticosteroid treatment completely resolved the symptoms.

**Conclusion:**

This is the first case of orbital myositis in a patient with discoid lupus erythematosus presenting with acute proptosis, diplopia and single extraocular muscle involvement.

## Introduction

Discoid lupus erythematosus (DLE) is a chronic cutaneous lupus erythematosus without internal organ involvement [1-3]. Ocular manifestations related to DLE are uncommon. Eyelid involvement [4,5], periorbital edema and erythema are described [6,7] and only two cases of orbital myositis (OM) have been reported [8,9]. We report a case of a Caucasian woman with a diagnosis of DLE based on a cutaneous skin biopsy who developed acute proptosis associated to orbital inflammation 5 years after the systemic diagnosis. She was not treated with hydroxychloroquine.

## Case presentation

A 37-year-old Caucasian woman affected by DLE presented to our hospital for a sudden onset of proptosis and bulbar pain of her right eye (RE). She also complained of blurred vision and diplopia. She was subjected to a complete ophthalmological examination. A Hertel exophthalmometer was used to value proptosis. A Hess screen test was performed to evaluate the diplopia. Endocrinological visit and thyroid function tests and blood test examination were prescribed. A cranial X-ray was performed to evaluate sinus inflammation. To evaluate the extraocular muscle involvement both ultrasound echography (Cinescan S Ophthalmic Ultrasound System) and orbital computed tomography (CT) were performed.

On the first examination, best corrected visual acuity (BCVA) was 20/32 in her RE and 20/20 in her left eye (LE). RE examination revealed bulbar position in adduction, marked limitation of her extraocular movements in the right position of gaze, swelling eyelids, scleral and episcleral injection and conjunctival chemosis. Hertel exophthalmometer values were 22mm in her RE and 18mm in her LE. Pupil response, intraocular pressure, and fundus examination were normal in both eyes. Her LE examination was normal. The endocrinologist according to the thyroid function test and neck echography excluded thyroid disease. A cranial X-ray showed transparency of the sinus. The blood analysis was normal. No leukopenia or thrombocytopenia was detected and only a slight increase in the velocity of her erythrocyte sedimentation rate (20mm/hour) was seen. The values of her creatine phosphokinase and complement levels were normal. An immunological profile showed high titer of antinuclear antibodies with a dotting pattern. No myositis-associated autoantibodies were detectable in the serum. According to the Score of Activity and Damage in DLE (SADDLE) [10], the systemic disease was classified not active.

**Figure 1:**
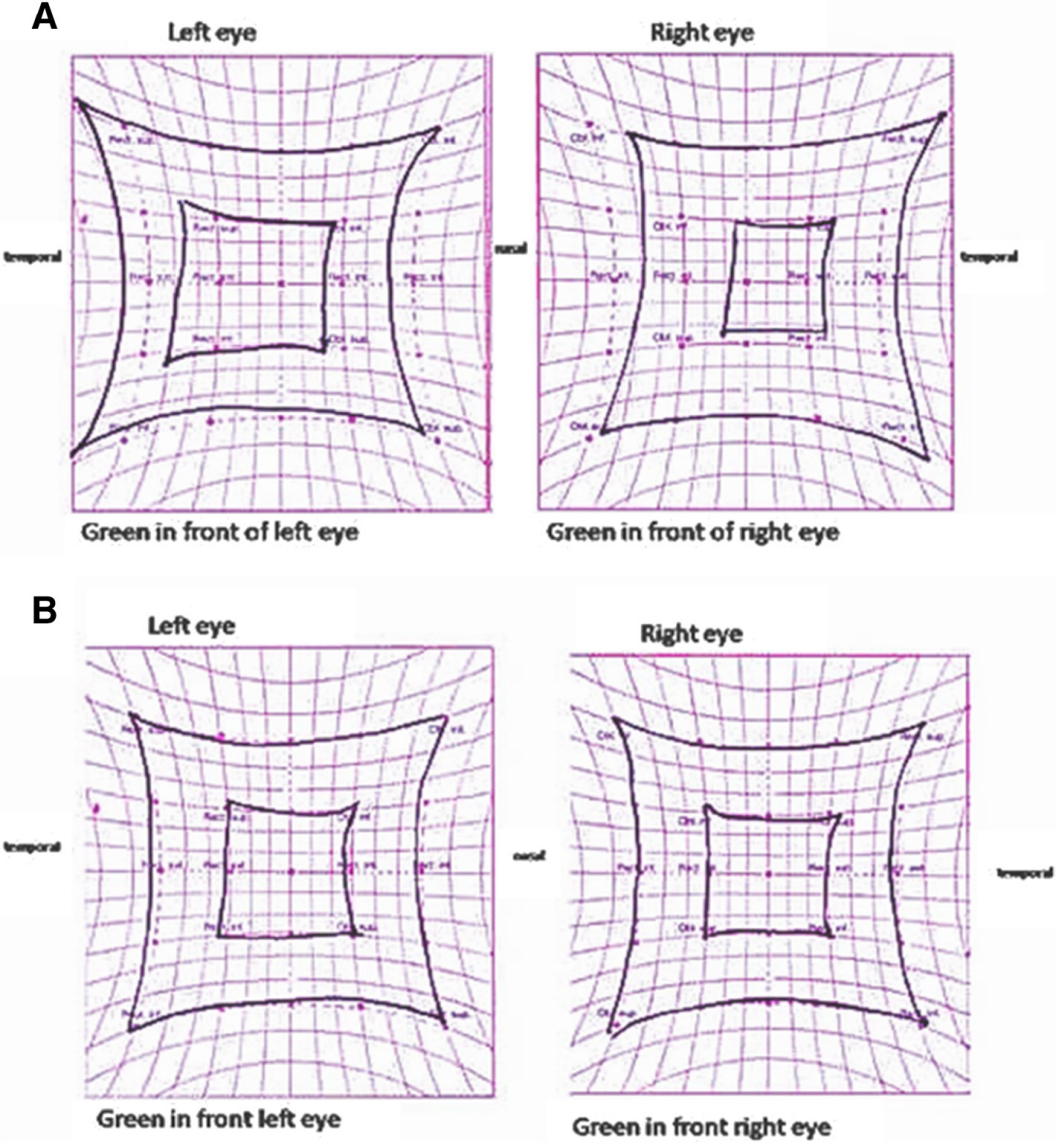
**Hess screen test.** Hess screen test at first visit **(A)** revealed a hypofunction of the right medial rectus with overreaction of the left lateral rectus. After 2 weeks of steroid treatment Hess test was normalized **(B)**.

**Figure 2:**
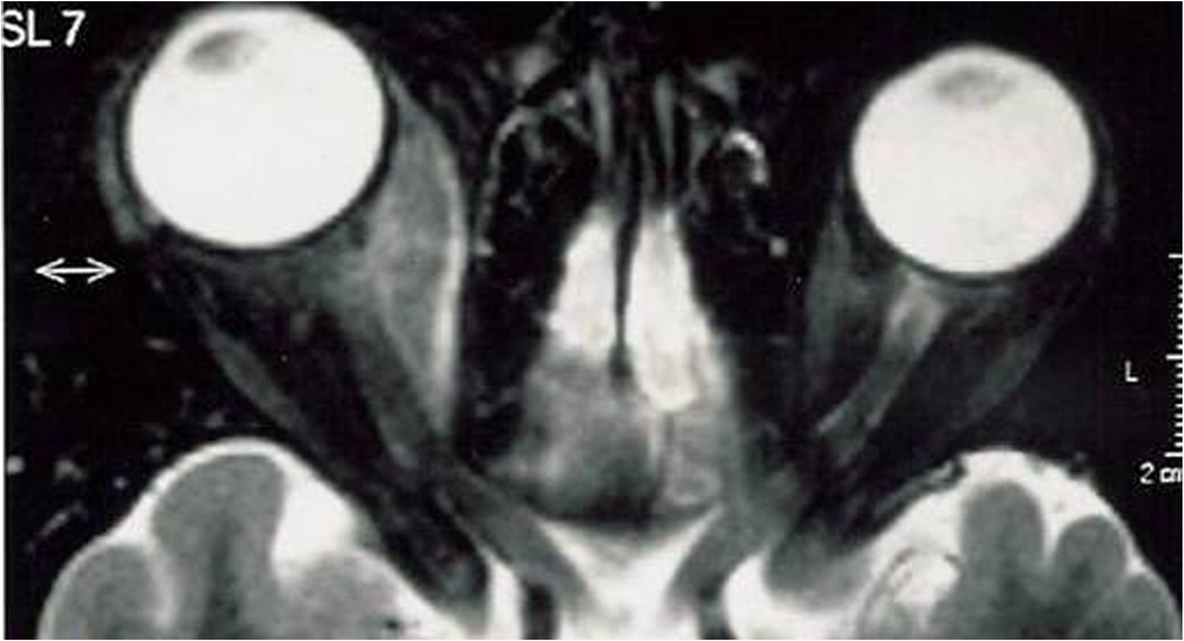
Computed tomography image showed enlargement of medial rectus muscle and proptosis in the right eye (bidirectional arrow).

A Hess screen revealed a hypofunction of the right medial rectus with overreaction of the left lateral rectus (Figure 1A). B-scan ultrasonography and orbital CT showed no orbital or periorbital mass, but an enlargement of the medial rectus muscle (7mm, maximum diameter; Figure 2). A diagnosis of OM was made based on the patient’s clinical features and abnormal CT findings. Treatment was initiated with three courses of intravenous methylprednisolone (1g/day) for 3 days followed by oral prednisolone (20mg/day); tapering the dosage 5mg per week. Her ocular pain rapidly improved within several days, and the diplopia and abnormal muscle findings markedly improved during the course of the steroid therapy. Her BCVA was 20/20 in both eyes, her intraocular pressure was also normal in both eyes. The measurements of proptosis using a Hertel exophthalmometer were 18mm in both eyes. Her Hess screen test was remarkably improved (Figure 1B). After 2 months, ultrasound echography showed a resolution of right medial muscle swelling. She developed an orbital inflammation recurrence with the same symptoms 6 months after finishing the steroid treatment. She was treated again with the same steroid protocol and the symptoms resolved. She was followed up for 2 years with no further relapses.

## Discussion

OM is a subtype of orbital inflammatory disease characterized by primary involvement of the extraocular muscle. The signs and symptoms of OM may also be seen in infection processes such orbital cellulites, neoplastic involvement for primary or metastatic orbital neoplasm, vascular disease such as carotid-cavernous fistulae, arteriovenous malformations, thrombosis, and autoimmune disease, the most common being thyroid eye disease. It is difficult to diagnose OM because there are no specific signs, symptoms, laboratory tests, or radiologic findings that can help in its diagnosis. In our case, orbital inflammation was characterized by the acute onset of painful proptosis and diplopia. CT showed the enlargement of single extraocular muscles, suggesting an inflammatory process that differs from thyroid eye disease ophthalmopathy where more muscles are enlarged. It is possible to observe the enlargement of only the medial muscle (Figure 2). The absence of temperature and the normal X-ray appearance of the sinus excluded orbital cellulites. An orbital biopsy was not indicated, considering the fast resolution with the corticosteroid therapy.

In the literature, only two case reports described the orbital inflammation associated to DLE but these cases were not associated with acute proptosis onset [8,9]. The first case [8] describes OM in a patient with DLE that was associated to subacute progressive ophthalmoplegia and pain with ocular movement. This patient received the corticosteroid treatment with a good outcome. The second report [9] describes a case of a woman with DLE who presented 2 months of slowly progressive painless periorbital swelling. CT imaging showed enlargement of superior and lateral rectus and lachrymal gland. She did not suffer diplopia. There was no improvement with systemic antibiotics or corticosteroids, but spontaneous resolution is described after discontinuing all treatment [9].

## Conclusions

This is the first case reported of OM in DLE presenting with acute onset monolateral proptosis, diplopia and the single extra ocular muscle involvement. Orbital inflammation syndrome should always be suspected in patients with DLE. Standard presentations of OM in patients with DLE are not described. An early imaging examination can be very helpful in the differential diagnosis of orbital inflammation; cooperation with dermatologists and rheumatologists is beneficial to the diagnostic and therapeutic treatment. The standard treatment is a steroid therapy but, in some cases, where the incidence of the relapse is very high, a new protocol including immunosuppressant or biological drugs will be evaluated [11].

## Consent

Written informed consent was obtained from the patient for the publication of this case report and any accompanying images. A copy of the written consent is available for review by the Editor-in-Chief of this journal.

## Abbreviations

BCVA: Best corrected visual acuity; CT: Computed tomography; DLE: Discoid lupus erythematosus; LE: Left eye; OM: Orbital myositis; RE: Right eye.

## Competing interests

The authors declare that they have no competing interests.

## Authors’ contributions

LA and RM designed the study, wrote the article and performed critical revision. AA and SB designed and conducted the study, collected and analyzed the data, wrote and reviewed the article. All authors read and approved the final manuscript.
